# Evaluation of abdominal and lumbar multifidus muscles thickness and relation to endurance, pain, fatigue and functional mobility in patients with Fibromyalgia syndrome: a case-control study

**DOI:** 10.1007/s00296-025-05813-7

**Published:** 2025-02-21

**Authors:** Yasemin Mirza, Fulden Sari, Pınar Diydem Yılmaz, Adem Küçük

**Affiliations:** 1 Faculty of Health Sciences, Department of Physiotherapy and Rehabilitation, Necmettin Erbakan University, Konya, Turkey; 2 Faculty of Physical Therapy and Rehabilitation, Department of Physiotherapy and Rehabilitation, Bingol University, Bingol, Turkey; 3 Faculty of Medicine, Department of Radiology, Necmettin Erbakan University, Konya, Turkey; 4 Faculty of Medicine, Department of Internal Medicine, Division of Rheumatology, Necmettin Erbakan University, Konya, Turkey

**Keywords:** Fibromyalgia, Abdominal muscles, Multifidus, Fatigue, Physical endurance, Ultrasonographic imaging

## Abstract

**Objective:**

The aim of present study was to compare abdominal (transversus abdominis (TrA), internal oblique (IO) and external oblique (EO)) and lumbar multifidus muscles (LM) evaluated with ultrasonographic (US) imaging in patients with FM (Fibromyalgia) and asymptomatic individuals and to examine the relationship between these muscle thickness and endurance, pain, fatigue and functional mobility.

**Methods:**

Women with FM group (n: 53, age: 45.96 ± 9.96 years), and asymptomatic control group (n: 49, age: 45.12 ± 7.28), were included in this study. Pain severity, disease activity, physical activity level, fatique, thickness of TrA, IO, EO and LM muscles and endurance, and functional mobility were evaluated with the Visual Analogue Scale (VAS), Fibromyalgia Impact Questionnaire (FIQ), International Physical Activity Questionnaire- Short Form (IPAQ-SF), Fatigue Severity Scale (FSS), US imaging, McGill core endurance tests, and physical fitness tests, respectively. FM patients were classified according to the FSS score.

**Results:**

The thickness of the IO (right side) (*p* = 0.013) and LM (both sides) (*p* < 0.001) muscles, lumbopelvic muscle endurance (all *p* < 0.001) and physical fitness tests (all *p* < 0.001) were lower in FM group compared to the asymptomatic group. No statistically significant differences were found in TrA, IO (left side), EO muscles thickness between the two groups (all *p* > 0.05). LM muscle thickness was significantly correlated with lumbopelvic muscle endurance (all *p* < 0.05), physical fitness tests (all *p* < 0.001) and fatique (*p* = 0.001). Moreover, significant differences in LM muscle thickness (*p* = 0.007), trunk flexor muscle endurance (*p* = 0.016), left trunk lateral flexor muscle endurance (*p* = 0.045) and 30-s chair stand test (*p* = 0.025) in favor of the low-fatigue group were detected.

**Conclusion:**

The thickness of LM muscle, lumbopelvik endurance and functional mobility in FM patients have been affected negatively. These findings should be considered in management of FM.

## Introduction

Fibromyalgia (FM) is a chronic syndrome described by persistent and generalized musculoskeletal pain. Patients also present with comorbid symptom such as fatigue, sleep disturbance, morning stiffness, cognitive problems and psychosomatic disorder [1]. In a recent review, the prevalence of FM is reported to be between 2% and 4% in the general population, with a higher frequency in women compared to men [2]. Although some factors are known to influence the pathophysiology of FM (such as genes, physical trauma and adverse life events), the etiology of FM is not clear [3]. It is thought that central sensitization to pain and impairments in endogenous pain inhibitory mechanisms play a crucial role at pathophysiology of FM [4]. As a sign of long-term physical inactivity, minor mitochondrial anomalies in muscle fibers and related muscle atrophies were reported in FM patients. Furthermore, muscle fiber type distribution, the involvement of antigravity muscles and energy needs can alter the rate of development of muscle atrophy and certain muscles may occur rapid atrophy [5]. These combined symptoms have a negative influence on functional ability and physical performance in patients with FM [6].

The lumbopelvic region is thought to be the centre of the kinetic chain and is also referred to as the ‘core region’. Anatomically, this region is surrounding by the abdominal muscles at the front, the gluteal, multifidus and paraspinal muscles at the back, the pelvic floor muscles at the bottom and the diaphragm muscle at the top [7]. The lumbopelvic muscles play an essential role in proprioceptive stimulation, postural stability and facilitating energy transfer between the upper and lower extremities. These muscles, often termed the “powerhouse,” stabilize the body and spine as the core of the kinetic chain. Their cocontraction, known as the “serape effect,” links upper and lower extremity stability. In this context, strengthening these muscles is essential for preventing injuries, rehabilitation, and enhancing performance [8]. Maintaining stability in the lumbopelvic region requires adequate strength, endurance, and coordinated co-activation of these muscles [9]. It may negatively affect the lumbopelvic muscles, is an important part of the body, in FM patients due to chronic pain, fatigue, cytokines and immobility [10, 11]. Previous research has documented that lumbopelvic muscle endurance is reduced in FM patients with compared to healthy individuals [11]. Mirza et al. further reported a correlation between kinesiophobia and lumbopelvic muscle endurance in FM patients [12]. Another recent study suggested that lumbopelvic muscle endurance is associated with upper and lower extremity functional level and pain in women with FM [13]. However, there are few studies investigating lumbopelvic muscles and related various parameters [11–13]. Therefore, thickness of the primary muscles supporting the lumbopelvic region in FM patients should be thoroughly investigated.

Ultrasound (US) has been recommended as a noninvasive, useful, accessible and safe method to measure muscle morphology and has been increasingly performed both in research and as a clinical tool throughout the rehabilitation process [14]. Insufficient study in the literature have demonstrated changes in muscle morphology by using US in FM patients. These include the assessment of neck, upper and lower extremity muscles [6, 15–17]. In many studies, physical performance and strength of patients with FM have been found to be lower than healthy individuals [16, 18, 19]. However, to our knowledge, there are insufficient studies in the literature evaluating the relationship between muscle thickness and physical performance in patients with FM [16].

Accordingly, lumbopelvic structure may negatively affect in FM patients; however, potential changes of lumbopelvic muscles in FM patients remain poorly understood. Therefore, the present study has focused on the abdominal (transversus abdominis (TrA), internal oblique (IO) and external oblique (EO)) and lumbar multifidus muscles (LM). Primarily; the current study was aimed to investigate abdominal and LM muscles thickness, lumbopelvic muscle endurance and functional mobility in FM patients and compare these healthy control. Secondarily; it was aimed to investigate relationship abdominal and LM muscles thickness and other parameters.

## Methods

### Study design

This case-control study was conducted based on the STROBE checklist for observational studies [20]. The Ethics Committee of Necmettin Erbakan University approved the study protocol (Approval Number: 2023/4699, Date: December 15, 2023). This study was conducted in regarding the rules of the Helsinki Declaration. It was performed in the Rheumatology Department of Necmettin Erbakan University Hospital between January 2024 and May 2024. All participants were informed about the detail of study and written informed consent was obtained.

### Participants

A total of 53 female FM patients aging from 18 to 65 years who presented at the Rheumatology Department of Necmettin Erbakan University Hospital and age-gender matched 49 asymptomatic control subjects participated in this study. Patients were included who had been diagnosed with FM by a rheumatologist at least one year prior regarding to the 2016 American College of Rheumatology diagnostic criteria [21]. Prior to the study, a detailed medical history of the patients was obtained and patients were excluded if they had neurological and/or other inflammatory rheumatic disorders, previous orthopedic or spinal surgery, serious spine deformity such as diagnosed scoliosis and/or any pathology involving the spine, any lumbal dysfunction (such as facet joint syndrome disc stenosis, spondylosis, herniation), serious cardiopulmonary problems such as chronic obstructive pulmonary disease, chronic heart failure, musculoskeletal problems related to lower extremities, vestibular system disorder, psychiatric disease, presence of malignancy, being pregnant, doing regular exercise (at least 150 min of moderate-intensity aerobic activity or 75 min of vigorous-intensity aerobic activity and at least two days of muscle-strengthening activities per week) [22], and not being volunteer. Asymptomatic individuals who having no low back pain, were not diagnosed with any musculoskeletal, neurologic, and/or rheumatologic disorders, not being pregnant, not doing regular exercise and being volunteer were participated in the control group.

### Outcome measures

All participants completed physical and demographic parameters as age, body mass index and smoking history. Then, disease-related characteristics of the patients were assessed by using Visual Analogue Scale (VAS) for pain, Fibromyalgia Impact Questionnaire (FIQ) for disease activity, International Physical Activity Questionnaire- Short Form (IPAQ-SF) for physical activity level, Fatigue Severity Scale (FSS) for fatique. In addition, detailed medical history such as current opioid use and FM duration were obtained. Finally, US measurements, lumbopelvic muscle endurance and physical fitness tests were evaluated for all participants. All these evaluations were performed by the same researcher and took 30–45 min. No negative situations was happened during the assessment process. Moreover, the patients were asked to not take any acute medication in the day before the evaluation.

### Primer outcomes

#### Ultrasound measurements

**Fig. 1:**
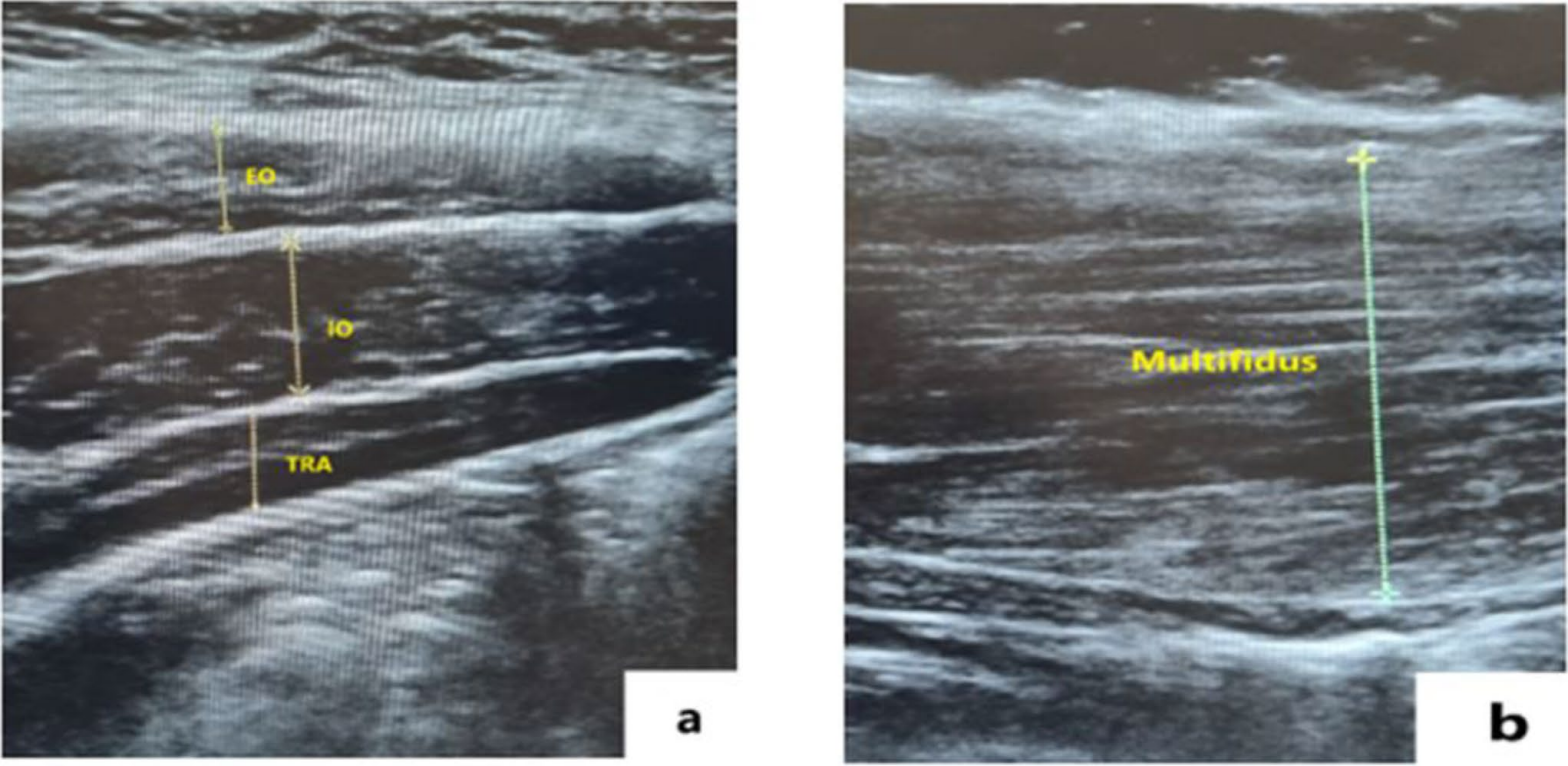
**(a)** Thickness of transverse abdominis, and external and internal oblique muscles, **(b)** Thickness of lumbar multifidus muscle

The thickness of the TrA, IO, EO and LM muscles were evaluated using US, performed by a qualified radiologist with a Siemens Acuson S3000 ultrasound system (Siemens Healthineers, Germany). A 9 MHz linear probe was employed to evaluate the TrA, IO and EO muscles. Participants were instructed to lie in a supine position on a stretcher and expose their abdomen. TrA, IO and EO muscles were imaged from both the right and left side, approximately 10 cm lateral and inferior to the umbilicus, using a 4–9 MHz linear probe, held at an oblique angle during scanning (Fig. 1a). During these measurements, participants were asked to avoid any actions that could influence the results, such as coughing or holding their breath, and were asked to maintain normal breathing. For the LM muscle, subjects were positioned in prone position. A 1.5-6 MHz convex probe was first placed transversely over the L4-S1 region to detect the L4-L5 intervertebral space. Once the L4-L5 level was fixed, the probe was aligned longitudinally along the spine (Fig. 1b). Thickness of the LM muscle on both the right and left sides was measured using this method [10].

### Seconder outcomes

#### Lumbopelvic muscle endurance

The endurance of the lumbopelvic muscles was assessed using the McGill core endurance tests, which consist of trunk flexion, extension, and right/left lateral flexion endurance tests. Previous researches have demonstrated excellent reliability for these tests, with intraclass correlation coefficients (ICC) reported as 0.97, 0.97, and 0.99 for trunk flexor, extensor, and right/left lateral flexor muscle endurance, respectively [23]. Participants were instructed on how to perform each position, followed by a trial to familiarize themselves with the postures. They were then encouraged to maintain each test position isometrically for as long as possible. The time held in the correct position was recorded in seconds.

#### Physical fitness tests

Timed Up and Go (TUG) test was performed to evaluate functional mobility. It has been performed in previous study involving the women FM population, showing excellent reliability (ICC = 0.935) [24]. Participants were asked to stand up from a chair without the use of armrests, walk a distance of 3 m as quickly as possible without running, turn and sit down again without utilizing the armrests. Time was recorded using a manual stopwatch by one of the researchers.

30-s chair stand test was performed to obtain lower body muscular strength. This test has been previously used in FM patients [13, 18]. It involves counting the number of times in 30 s that participant may rise from a sitting position to a full stand.

#### Pain severity

Self-reported pain severity of patients was evaluated using VAS. This scale consists of a line, which is defined 0: No pain, 10: Unbearable pain. Patients described their pain severity on a line [25].

#### Disease activity

Disease activity of patients was assessed using FIQ. It consists of ten distinct parameters, which include daily activities, difficulties in performing occupational tasks, fatigue, morning stiffness, pain levels, as well as anxiety and depression. With higher scores reflecting greater disease impact and activity, the total score ranges from 0 to 100 [26].

#### Physical activity level

Physical activity level of the patients were assessed by using IPAQ-SF. The IPAQ-SF assesses energy expenditure over the past week in terms of total metabolic equivalent. Moderate and vigorous activity and the total frequency (days) and duration (minutes) of walking are taken into account in the calculation of the total score. For an activity to be contributed in total score, each activity should be done for a minimum duration of 10 min at a time. According to total score, patients are classified as the follows; low physical activity level: 600 MET-min/week or lower, moderate physical activity level: 601–3000 MET-min/week and high physical activity level: more than 3000 MET-min/week [27].

#### Fatigue

FSS, which is a 9-item, was performed to assess the fatigue severity of patients. FSS consists of likert-type scoring, and each item is rated on a scale from 1 (completely disagree) to 7 (completely agree). The total score is calculated by summing all individual scores and dividing the result by 9. A cut-off score of 4 has been established; a total score of 4 or higher presents a high level of fatigue, while a score below 4 presents a low level of fatigue [28]. In this study, patients were divided into two groups: those with a fatigue score of 4 or higher were assigned to the high-fatigue group, while the remaining patients were categorized into the low-fatigue group.

### Statistical analysis

Statistical analyses were conducted using SPSS Version 22.00 (SPSS Inc., Chicago, IL) and Graphpad Prism version 8.3.0 (Graphpad Software). Visual assessments (histograms/probability graphs) and analytical tests (Kolmogorov-Smirnov/Shapiro-Wilk) were used to determine whether the variables were normally distributed. Continuous variables are presented as mean and standard deviation (SD) or as median (minimum–maximum), while categorical variables are expressed as frequencies and percentages. Comparisons between groups were conducted using either the Independent Samples t-test or the Mann-Whitney U test, depending on the data distribution.

Correlation analyses were conducted using Spearman method, with interpretation as follows: negligible (0–0.29), low (0.30–0.49), moderate (0.50–0.69), high (0.70–0.89), and very high (0.90–1.00) [29]. Cohen’s guidelines were performed to evaluate the magnitude of a correlation for interpretation purposes. Based on Cohen’s classification, a d value below 0.2 signifies a negligible effect, whereas a value greater than 0.2 suggests a small effect. Similarly, a d value exceeding 0.5 denotes a moderate effect, while a value greater than 0.8 indicates a large effect. Lastly, a d value exceeding 1.13 represents a very large effect [30]. P value < 0.05 was accepted for statistical significance level.

In the absence of comparable researches on this issue, a pilot study was conducted with ten participants from each group. The effect size of this preliminary study, based on the IO muscle thickness measurements, was calculated to be 0.66. Consequently, a total of 98 participants, with at least 49 in each group, was found sufficient to achieve 90% statistical power, assuming an effect size of d = 0.66, α = 0.05 (type I error), and β = 0.10 (type II error). The required sample size was determined using the G*Power 3.1.9.2 software.

## Results

### Characteristics of the study groups

**Fig. 2:**
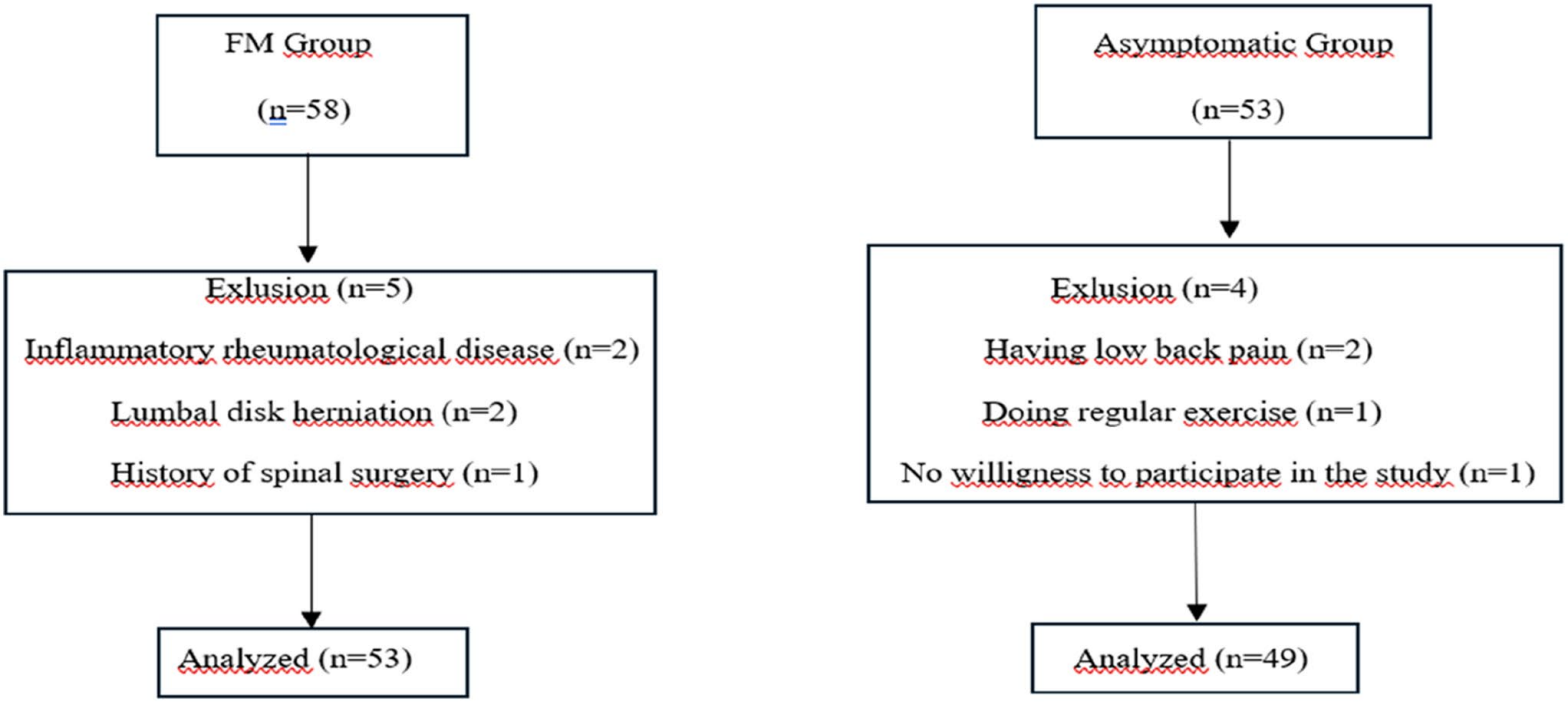
Flow chart of the participants

In total, 111 participants were initially evaluated for the study. Fifty-three patients in the FM group and 49 participants in the asymptomatic group completed the study.The details regarding included and excluded participants are showed in a flowchart (Fig. 2). FM and the control group were comparable regarding physical and demographic characteristics (*p* > 0.05, Table 1). Clinical characteristics of the patients were as follows; the median disease duration was 5 (1–25) years, and the median VAS score was 8 (3–10). Thirty-five (66%) patients had been using analgesic medicine. Detailed clinical characteristics of patients are listed in Table 1.

**Table 1:** Physical and demographic characteristics and clinical parameters of groups

Parameters	FM group (*n* = 53)	Control group (*n* = 49)	*p*-value
**Physical and demographic characteristics**			
Age (years)	45.96 ± 7.96	45.12 ± 7.28	0.581^a^
BMI (kg/m^2^)	28.77 (21.09–49.78)	29.37 (22.64–32.44)	0.451^b^
Current smoker, yes/no (n)	6/47	5/44	0.907^a^
**Clinical parameters**			
Disease duration (years)	5 (1–25)	-	-
Painkiller use, n (%)	35 (66)	-	-
Pain (VAS)	8 (3–10)	-	-
FIQ (score)	52.46 ± 12.16	-	-
IPAQ (MET-min/wk)	198 (0-819)	-	-
FSS (score)	4.88 (1.66–6.44)	-	-

^a^Independent sample t test, ^b^Mann–Whitney U test

Variables are presented as mean ± standard deviation, median (min-max) and n (%)

n: Number, BMI: Body-mass index, MET: Metabolic equivalent, FM: Fibromyalgia, VAS: Visual Analog Scale, FIQ: Fibromyalgia Impact Questionnaire, IPAQ: International Physical Activity Questionnaire, FSS: Fatigue Severity Scale

### Comparative analysis of the study groups

Comparative data of the study groups in terms of the thickness of TrA, IO, EO and LM muscles, lumbopelvic endurance and physical fitness tests are provided in Table 2. In FM group, the thickness of the IO (right side) and LM (both sides) muscles were lower than that of the control group (*p* < 0.05). No significant difference was observed between the groups in terms of other muscles thickness (*p* > 0.05). All lumbopelvic muscle endurance test results for the FM group were significantly lower compared to those of the control group (*p* < 0.001). When the physical fitness tests were examined, FM group demonstrated lower levels of physical performance in the TUG and 30-s chair stand tests compared to the the control group (*p* < 0.001, Table 2).

**Table 2:** Comparison of the thickness of abdominal and LM muscles, lumbopelvic endurance and physical fitness tests of the groups

Parameters	FM group (*n* = 53) Median (min-max) Mean ± SD	Control group (*n* = 49) Median (min-max) Mean ± SD	*p*-value	Effect size (d)
External Oblique (mm)				
- R	4.75 ± 1.06	4.93 ± 0.96	0.385^a^	0.18
- L	4.68 ± 0.98	4.84 ± 1.05	0.437^a^	0.16
Internal Oblique (mm)				
- R	4.7 (3–7.8)	5 (3.1–8.8)	**0.013** ^*b^	0.60
- L	5.1 (2.6–7.9)	4.95 (3–11)	0.603^b^	0.23
Transversus Abdominis (mm)				
- R	3.85 ± 0.78	3.89 ± 0.96	0.801^a^	0.04
- L	3.51 ± 0.84	3.83 ± 0.9	0.075^a^	0.36
Lumbar Multifidus (mm)				
- R	11.7 (7.6–15.1)	16.25 (11.7–28.3)	**< 0.001** ^**b^	1.97
- L	11.7 (7.3–14.9)	16.4 (12.1–29.1)	**< 0.001** ^**b^	2.10
**Lumbopelvic muscle endurance**				
Trunk flexor muscle endurance test (sec)	8.73 (0–30)	28.01 (8.06–36)	**< 0.001** ^**b^	2.68
Trunk extensor muscle endurance test (sec)	8.92 (0–26.69)	30 (24.67–35.68)	**< 0.001** ^**b^	4.39
Right trunk lateral flexor muscle endurance (sec)	10.5 (0–30)	30 (20.2–35)	**< 0.001** ^**b^	2.90
Left trunk lateral flexor muscle endurance test (sec)	10.37 (0–30)	30 (10.1–36)	**< 0.001** ^**b^	2.90
**Physical Fitness Tests**				
TUG (sec)	7.86 (5.9–18.08)	5.24 (4.35–5.92)	**< 0.001** ^**b^	2.31
30-s chair stand test (rep)	11 (5–16)	16 (14–22)	**< 0.001** ^**b^	2.68

^a^Independent sample t test, ^b^Mann–Whitney U test

M: mean, SD: standard deviation, Min–max: minimum–maximum

LM: Lumbar Multifidus, FM: Fibromyalgia, TUG: Timed Up and Go

Bold values represent statistically significant results (*p < 0.05, **p < 0.001)

### Correlation analysis

Correlation analyses between the thickness of TrA, IO, EO and LM muscles and other study outcomes are indicated in Table 3. The EO muscle thickness had poor correlation with right/left trunk lateral flexor muscle endurance and TUG test (*p* < 0.05). Also, the IO muscle thickness was associated with trunk flexor muscle endurance, right trunk lateral flexor muscle endurance and physical fitness tests (*p* < 0.05). The TrA muscle thickness had poor correlation with trunk flexor muscle endurance (*p* < 0.05). Moreover, the highest relationship was detected between LM muscle thickness and all lumbopelvic muscle endurances, physical fitness tests, fatique (*p* < 0.05, Table 3).

**Table 3:** Correlations between the thickness of abdominal and LM muscles, lumbopelvic endurance and physical fitness tests and clinical features in FM patients

*n*: 53	External oblique	Internal oblique	Transversus abdominis	Lumbar multifidus
	rho p	rho p	rho p	rho p
Trunk flexor muscle endurance	0.270	0.368	0.272	0.429
	0.051	0.007*	0.049*	0.001*
Trunk extensor muscle endurance	0.215	0.250	0.249	0.389
	0.122	0.071	0.072	0.004*
Right trunk lateral flexor muscle endurance	0.297	0.319	0.111	0.394
	0.031*	0.020*	0.429	0.003*
Left trunk lateral flexor muscle endurance	0.292	0.266	0.082	0.384
	0.034*	0.054	0.559	0.004*
TUG	-0.205	-0.268	-0.101	-0.754
	0.040*	0.007*	0.313	< 0.001**
30-s chair stand test	0.158	0.282	0.030	0.685
	0.115	0.004*	0.768	< 0.001**
Pain	-0.137	-0.025	0.094	0.019
	0.327	0.861	0.504	0.892
FIQ	-0.019	-0.163	0.033	-0.209
	0.890	0.244	0.812	0.133
IPAQ	0.160	0.033	0.033	0.088
	0.253	0.812	0.812	0.532
FSS	-0.086	-0.039	-0.039	-0.426
	0.538	0.780	0.780	0.001*

rho: Spearman’s rank correlation coefficient

LM: Lumbar Multifidus, FM: Fibromyalgia, TUG: Timed Up and Go, FIQ: Fibromyalgia Impact Questionnaire, IPAQ: International Physical Activity Questionnaire, FSS: Fatigue Severity Scale

Bold values represent statistically significant results (*p < 0.05, **p < 0.001)

### Comparative analysis of the patients with high- and low-fatigue

When comparing the thickness of TrA, IO, EO and LM muscles, lumbopelvic endurance, and physical fitness test results between the high-fatigue and low-fatigue groups among FM patients, significant differences were observed in LM muscle thickness, trunk flexor endurance, left trunk lateral flexor endurance, and 30-second chair stand test performance, all favoring the low fatigue group (*p* < 0.05). No other significant differences were identified between the groups (*p* > 0.05) (Table 4).

**Table 4:** Comparison of the thickness of abdominal and LM muscles and lumbopelvic endurance and physical fitness tests between high- fatigue and low-fatigue groups

	Low-fatigue group (*n* = 31)	High-fatigue group (*n* = 22)	*p*-value	Effect size (d)
	Median (min-max)	Median (min-max)		
External Oblique (mm)	4.9 (3.5–6.7)	4.25 (2.2–7.2)	0.122	0.46
Internal Oblique (mm)	4.8 (3–6.2)	4.4 (3.2–7.8)	0.432	0.03
Transversus Abdominis (mm)	3.9 (2.3–6.8)	3.6 (2.6–6.3)	0.878	0.04
Lumbar Multifidus (mm)	12.4 (10.1–15.1)	11.45 (7.6/13.8)	0.007*	0.89
**Lumbopelvic muscle endurance**				
Trunk flexor muscle endurance test (sec)	9.33 (3.02–30)	6.32 (0–21.46)	0.016*	0.63
Trunk extensor muscle endurance test (sec)	8.92 (3.36–26.69)	8.35 (0–18.85)	0.149	0.45
Right trunk lateral flexor muscle endurance (sec)	11.39 (0–30)	8.22 (0–30)	0.106	0.47
Left trunk lateral flexor muscle endurance test (sec)	11.01 (0–30)	8.88 (0–30)	0.045*	0.55
**Physical Fitness Tests**				
TUG (sec)	7.86 (5.9–9.9)	8.03 (6.89–18.08)	0.081	0.54
30-s chair stand test (rep)	12 (5–16)	10.5 (8–15)	0.025*	0.52

Mann–Whitney U test, Min–max: minimum–maximum

LM: Lumbar Multifidus, TUG: Timed Up and Go

Bold values represent statistically significant results (*p < 0.05)

## Discussion

To the best of our knowledge, this is the first study to comprehensively evaluate female patients with FM concerning abdominal and LM muscles thickness, endurance and functional mobility. With this new aspects, the main findings of the present study revealed that women with FM patients had less IO and LM muscle thickness, lumbopelvic endurance and functional mobility compared to asymptomatic women. It also showed that the LM muscle thickness were associated with lumbopelvic muscle endurance, functional mobility and fatique. Moreover, FM patients, who had lower fatique level, had more LM muscle thickness, trunk flexor muscle endurance and left trunk lateral flexor muscle endurance and functional mobility.

The lateral abdominal wall, i.e., the TrA, IO, and EO muscles, together with the LM, play a crucial role in stabilizing the lumbar spine while also supporting balance and posture. A decrease in lumbopelvic muscles size could adversely affect their structure and functionality [31]. Studies assessing the relationship between the morphological structure of different muscles and clinical features in patients with FM are limited. Kuzu and Aras reported significantly reduced cervical extensor muscle thickness in FM patients compared to healthy individuals, with a negative correlation observed between cervical extensor thickness and functional status [15]. On the contrary, Valera-Calero et al. revealed that no association between the cervical multifidus muscle morphology and clinical characteristics such as pain intensity and disease activity FM patients [17]. Balaban et al. reported that patients with fibromyalgia and migraine had decreased longus colli muscle cross-sectional area and increased upper trapezius muscle stiffness compared to patients with fibromyalgia but no migraine [32]. Another study findings stated that the trapezius, upper arm and gastrocnemius medialis/lateralis muscle thicknesses in FM patients significantly decreased compared with the control group [16]. Differences in methodology, such as variations in ultrasound imaging techniques, the heterogeneity of FM patients including variations in disease duration, severity, comorbidities, and physical activity levels may contribute to these discrepancies. These conflicting findings highlight the variability in muscle morphology and associated factors in FM patients. In line with this findings, present study particularly focused on the thickness of abdominal and LM muscles, which are important in spine health, in patients with FM. We found that especially LM muscle thickness in patients with FM was lower compared to those control group. This finding suggests that the deep lumbar extensor muscles may be more vulnerable to alterations due to generalized pain and trigger points, especially in the back region in patients with FM [33], while no differences were found in abdominal muscles thickness. In consistent with our result, some evidence suggests that no significant difference regarding to abdominal muscle thickness between healthy subjects and those patients with ankylosing spondylitis [31]. Another likely reason explaining our findings could be current evidence of the role of LM muscle in spinal stability and control [34]. Due to the frequent axial pain in FM and intense trigger points in back region, inhibition of neural control in LM muscle occurs. As a result, the LM muscle cannot perform their functions effectively, which most likely debilitates postural control. This mechanism becomes chronic and potentially leading to atrophy of the LM [35, 36]. Considering that postural instability is increasingly observed in FM patients [37], specific stabilization exercises might be effective in improving LM muscle function in women with FM.

Some evidence suggests that the decrease in lumbopelvic muscle endurance in various populations may be due to atrophy of the lumbar region muscles [10, 38]. Several studies have demonstrated that common symptoms in patients with FMS, including chronic pain, fatigue, reduced mobility, and elevated cytokine levels, may lead to muscle atrophy. These studies typically evaluate grip strength as well as strength and endurance in both the upper and lower extremities [39, 40]. There is also a significant evidence gap regarding the evaluation of lumbopelvic muscle endurance in FM patients. Toprak Celenay et al. stated that lumbopelvic muscle endurance is reduced in patients with FM compared to healthy individuals [12]. Another study indicated that lumbopelvic muscle endurance was associated with kinesiophobia in FM patients [13]. Similar to these studies, we found lumbopelvic muscle endurance was substantially decreased in the patients with FM compared to healthy subjects. One should take into account that the lumbopelvic muscles play a crucial role in maintaining physical function and balance in daily activities, as well as in supporting spinal health [41, 42]. We, therefore, evaluated physical fitness tests in FM patients. Previously, it has been documented that physical impairment in FM patients may reduce their ability to carry out daily living activities [19]. In this regard, our findings revealed that the FM patients apparently exhibited lower performance in the TUG and the 30-second chair stand tests compared to the healthy individual, which is in line with previous researches [19, 43]. In fact, most of our patients had low level physical activity. Moroever, it is possible that a physically inactive sedentary lifestyle may lead to decreased lumbopelvic muscle endurance and functional mobility in FM patients. Based on the current findings, patients with FM should be adviced for strengthening and endurance exercises and physical activity counseling.

In literature, there was no evidence for an association between lumbopelvik muscle endurance, functional mobility, clinic paremeters and changes in lumbopelvic muscle morphology in FM patients. Our findigs yielded that especially LM muscle thickness was related to lumbopelvic muscle endurance, TUG, the 30-second chair stand tests and fatique. It is noteworthy, however, that we did not find any correlation between EO, IO, TrA muscles thickness and lumbopelvic muscle endurance and other parameters. One possible reason is that muscle endurance may not only depend on muscle thickness. Muscle endurance is related to functional capacity, neuromuscular control, and the muscle’s ability to efficiently use energy [44]. An another possible explanation for these results could be that the changes in LM muscle morphology is greater in FM patients than in abdominal muscles. Some evidence suggests that the LM muscle, a key muscle for trunk stabilization, may experience reduced activity within 24 h of acute back pain onset, with subsequent morphological changes localized during the subacute phase and becoming more prevalent in chronic disease stages [45]. Moreover, long-term pain, immobility and functional impairment have been reported to cause atrophy of the lumbar muscles. In patients with chronic back pain, decreased LM muscle length and fat content, which is the most important supporter of the vertebral column, have been observed [46]. These results indicate that there is muscle atrophy in LM muscles in FM patients and LM muscle associated with functional status. Thus, we suggest that core and stability exercises are important in this patient group.

Fatigue is markedly high in FM patients. Up to 82% of patients with FM describe severe fatigue [47] and more than 25% identify fatigue as their main complaint [48]. Previous study demonstrated that fatigue is closely aasociated with functional capacity in daily activities and physical deconditioning in patients with FM [49]. We obtained a negative correlation between LM muscle thicknesses and fatigue level. Furthermore, patients in the low-fatigue group were found to have greater LM muscle thickness, lumbopelvic muscle endurance and lower body muscular strength compared with patients in the high fatigue group. In this content, it may be beneficial to enhance the core muscles to deal with the negative influences of fatigue. Nevertheless, future research is needed to determine the most effective program.

This is the first study to comprehensively assess the thickness of spesific lumbopelvic muscles (TrA, IO, EO and LM), endurance, and functional mobility, in women with FM patients. It is well known that lumbopelvic muscle endurance is decreased and postural stability is impaired in FM patients [11, 37]. However, properties of lumbopelvic muscles in patients with FM remains unclear. Our study provides new insights on the understanding of abdominal and LM muscles features and relationship between these muscle thickness and lumbopelvic endurance, functional mobility and fatique in FM patients. However, the results of the present study should be taken into account in light of its limitations. Firstly, the findings may reflect pure associations, and a cause effect relationship should not be assumed. Longitudinal studies are recommended to investigate causality, which is not possible given the cross-sectional nature of this study. Secondly, we did not perform routine diagnostic imaging, such as MRI or X-ray, in all participants to exclude musculoskeletal disorders in the lumbopelvic region. Instead, exclusion was based on participants’ medical history, clinical examination, and available previous imaging results. While this approach is consistent with standard clinical practice, it is possible that some underlying musculoskeletal conditions may not have been detected in participants who neither reported symptoms nor underwent prior imaging. This may have influenced the results, especially the significant differences in the indicators of the right and left muscles. Thirdly, the findings could not be generalized to all FM patients, since we conducted this study with only female. Future studies are recommended to include both sexes and diverse populations. In addition, we evaluated only TrA, IO, EO and LM muscles thickness. Further studies should comprehensively assess the muscle morphology such as muscle mass, muscle density and muscle diameter and other lumbopelvic muscles such as diaphragm, iliolumbar and rectus abdominus muscles. While, muscle morphology is well known to be influenced by factors such as physical activity levels, fear of movement, and body composition [50]. Future studies can investigate lumbopelvic muscle morphology in FM patients at different physical activity levels. The relationship between spinal alignment, postural stability and lumbopelvic muscle thickness should also be examined. The need for standardized measurement protocols and more detailed subgroup analyses in future studies to better understand the factors influencing muscle morphology in FM patients. Finally, future intervention studies can explore the effectiveness of a core stabilization exercise on lumbopelvic muscle thickness in FM patients.

In conclusion, our findings suggest the thickness of LM and IO muscles and lumbopelvic endurance and functional mobility in female with FM compared to asymptomatic female were affected negatively. It was also observed that the decrease in LM muscle thickness had a negative effect on lumbopelvic muscle endurance, functional mobility and fatique. We believe that our findings will contribute to the limited understanding about the lumbopelvic structure in patients with FM and provide useful information for the design of personalized structured exercise programs for FM patients. In the treatment of women with FM, the lumbopelvic muscle thickness, endurance, and functional mobility changes should be taken into account in clinics.

## Data Availability

Data sharing is open upon request.
